# 3D Graphene Oxide-Polyethylenimine Scaffolds for Cardiac Tissue Engineering

**DOI:** 10.1021/acsami.3c00216

**Published:** 2023-03-07

**Authors:** Serena Pilato, Samanta Moffa, Gabriella Siani, Francesca Diomede, Oriana Trubiani, Jacopo Pizzicannella, Daniele Capista, Maurizio Passacantando, Paolo Samorì, Antonella Fontana

**Affiliations:** † Dipartimento di Farmacia, Università “G. d’Annunzio” di Chieti-Pescara, Via dei Vestini, 66100 Chieti, Italy; ‡ Dipartimento di Tecnologie Innovative in Medicina & Odontoiatria, Università “G. d’Annunzio” di Chieti-Pescara, Via dei Vestini, 66100 Chieti, Italy; § ASL02 Lanciano-Vasto-Chieti, Ospedale “Ss. Annunziata”, 66100 Chieti, Italy; ∥ Dipartimento di Scienze Fisiche e Chimiche, Università degli Studi dell’Aquila, Via Vetoio, 67100 Coppito, L’Aquila, Italy; ⊥ Université de Strasbourg, CNRS, ISIS, 8 alleé Gaspard Monge, 67000 Strasbourg, France; # UdA—TechLab, Research Center, Università “G. d’Annunzio” di Chieti-Pescara, Via dei Vestini, 66100 Chieti, Italy

**Keywords:** graphene oxide, polyethylenimine, atomic force microscopy, X-ray photoelectron spectroscopy, three-dimensional nanomaterials, cardiac muscle HL-1 cells

## Abstract

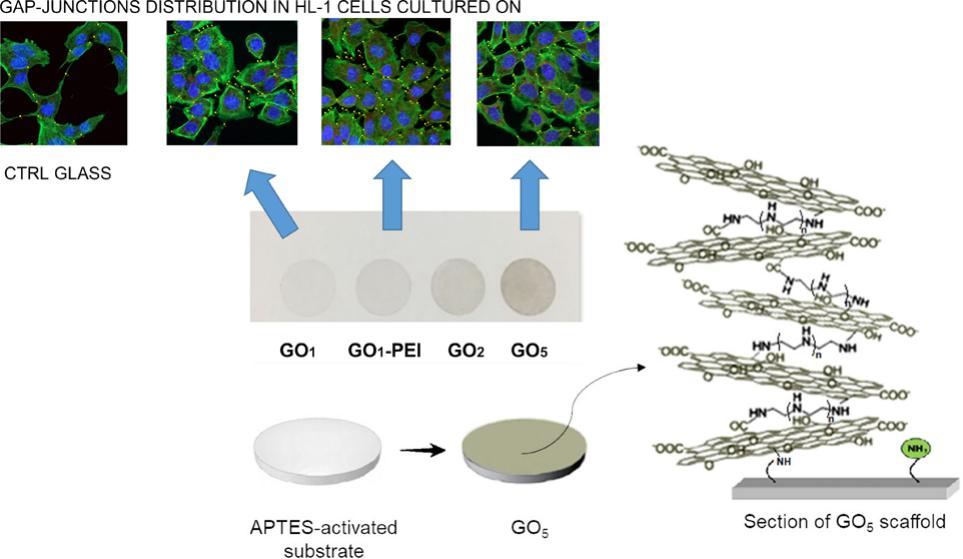

The development of novel three-dimensional (3D) nanomaterials combining high biocompatibility, precise mechanical characteristics, electrical conductivity, and controlled pore size to enable cell and nutrient permeation is highly sought after for cardiac tissue engineering applications including repair of damaged heart tissues following myocardial infarction and heart failure. Such unique characteristics can collectively be found in hybrid, highly porous tridimensional scaffolds based on chemically functionalized graphene oxide (GO). By exploiting the rich reactivity of the GO’s basal epoxydic and edge carboxylate moieties when interacting, respectively, with NH_2_ and NH_3_^+^ groups of linear polyethylenimines (PEIs), 3D architectures with variable thickness and porosity can be manufactured, making use of the layer-by-layer technique through the subsequent dipping in GO and PEI aqueous solutions, thereby attaining enhanced compositional and structural control. The elasticity modulus of the hybrid material is found to depend on scaffold’s thickness, with the lowest value of 13 GPa obtained in samples containing the highest number of alternating layers. Thanks to the amino-rich composition of the hybrid and the established biocompatibility of GO, the scaffolds do not exhibit cytotoxicity; they promote cardiac muscle HL-1 cell adhesion and growth without interfering with the cell morphology and increasing cardiac markers such as Connexin-43 and Nkx 2.5. Our novel strategy for scaffold preparation thus overcomes the drawbacks associated with the limited processability of pristine graphene and low GO conductivity, and it enables the production of biocompatible 3D GO scaffolds covalently functionalized with amino-based spacers, which is advantageous for cardiac tissue engineering applications. In particular, they displayed a significant increase in the number of gap junctions compared to HL-1 cultured on CTRL substrates, which render them key components for repairing damaged heart tissues as well as being used for 3D in vitro cardiac modeling investigations.

## Introduction

Myocardial infarction is associated with significant cell death and loss of heart functions. Therefore, it represents one of the leading causes of mortality worldwide.^1^ Since myocardial tissue lacks autoregenerative capacity, the damage induced by cardiac injury is permanent, and treatment options are limited.^1^ Treatments may involve medical management, change of lifestyle behaviors, and use of cardiac devices, with the aim of reducing symptoms, slowing disease progression, and ultimately reducing mortality. In the field of tissue engineering, the development of highly organized and functional three-dimensional (3D) complex scaffolds is highly relevant since native tissues and organs exhibit 3D complex architectures composed of extracellular matrix (ECM), different cell types, and chemical and physical signaling pathways. Efforts in cardiac tissue engineering strategies have been focused on the development of biomaterials that can mimic the myocardium extracellular matrix and promote cell growth and organ repair.^2^ Biomaterials currently used in cardiac tissue engineering must meet precise requirements, such as substrate stiffness and flexibility, biocompatibility, and pore size able to allow cell and nutrient infiltration. These features have been addressed in the design of first-generation biomaterials such as decellularized matrix and biomaterials based on collagen, fibrin, chitosan, gelatin, and alginate to name a few. The poor electrical conductivity of the above-mentioned biomaterials has been overcome by incorporating conductive components such as metals, electroconductive polymers, and carbon-based nanomaterials.^3^

Nanosized carbon materials, including carbon nanotubes,^4^ graphene-based nanosheets,^5^ carbon nanohorns,^6^ and carbon nanofibers,^7^ have gathered significant attention for tissue engineering applications because of their mechanical and electrical properties. Indeed, carbon-based materials have been regarded as valuable components to be incorporated into nonconductive or low conducting materials yielding scaffolds with higher physical strength, biological activity, and conductivity, which ultimately can direct cells to form electrically conductive networks.^3^ Moreover, the natural propensity of graphene-based nanostructures to undergo self-assembly has been widely explored to build up “all-carbon” three-dimensional scaffolds for application in nanomedicine.^8,9^ In the last decade, different graphene-based biomaterials have been investigated, demonstrating that the properties of the produced scaffold can vary tremendously as a result of the chosen preparation methodology,^10−12^ surface morphology,^13^ type of graphene exploited,^14^ its chemical functionalization,^15^ defects,^16^ and environmental conditions/stimulations.^17^ Since many factors and parameters are involved, the engineering or design of the scaffold of interest has to be carefully studied to ensure reproducibility and fine tuning of the properties in view of the desired application.^10,18^ Among the graphene-based materials, graphene oxide (GO) represents the “hydrophilic derivative” of graphene, and it is usually preferred over graphene for producing homogeneous aqueous suspensions due to the oxygen-containing functional groups on its basal plane (hydroxyl and epoxide groups) and edges (carboxyl groups).^19,20^ GO exhibits a relatively low electrical conductivity compared to graphene sheets, but it has been shown that GO can support attachment, growth, and differentiation of cells with little or no cytotoxic effects.^21−23^ Three-dimensional GO foams have been prepared via self-assembly of reduced GO and nanohydroxyapatite composites for tissue engineering applications.^24^ Similarly, rolled graphene oxide foams, prepared by sedimentation of GO sheets, have been investigated for the differentiation of stem cells and regeneration of nervous systems.^25^

In this work, a three-dimensional scaffold comprising GO flakes was developed for cardiac repair applications. To obtain a 3D porous structure and to improve the biological activity of GO, the scaffolds have been prepared by step-wise covalent growth of multilayer architectures of GO sheets alternated by linear polyethylenimine (PEI) macromolecules, the latter acting as linkers and spacers. Cross-linking between GO and PEI has been previously reported to form structurally defined foams with controlled porosity,^26,27^ adsorbing sponge materials,^28^ and GO framework membranes for ion-selective separations.^29^ Here, we used for the first time such a cross-link synthetic procedure at interfaces by means of the covalent layer-by-layer (LbL) technique^30^ to generate controlled 3D architectures. In this way, we can take advantage of the powerful LbL methodology,^31^ by assembling different components through the joint effect of noncovalent (i.e., electrostatic, π–π interactions, or hydrogen bonds) and covalent interactions, to obtain scaffolds combining programmed structures, compositions, and robustness. Compared to the bulk preparation,^32^ this technique allows us to (i) control the thickness of the substrate by varying the number of alternative immersions; (ii) choose at will the composition of the external layer, being either GO or PEI, and (iii) simplify the rinsing steps during the preparation process to attain higher compositional and structural control. The morphology of these scaffolds is key to promoting the growth and the alignment of the electroactive cardiac cells in one direction, mimicking the anisotropic alignment of cardiomyocytes present in the cardiac tissue.^33^ The scaffolds were manufactured by using 3-(aminopropyl)triethoxysilane (APTES)-activated substrates (i.e., round glass coverslips or SiO_2_ wafer), subsequently dipped in aqueous solutions of GO and PEI, leading to the formation of 3D networks with a number of alternate GO and PEI layers ranging from 1 to 5. Morphological insights into the GO-based 3D networks were obtained by atomic force microscopy (AFM) and scanning electron microscopy (SEM) investigations, whereas Young’s modulus of the 3D networks was measured by the peak force QNM mode of AFM. Moreover, the elemental and chemical properties of the scaffolds were studied by X-ray photoelectron spectroscopy (XPS) and water contact angles (CAs). The expression of cardiac markers such as Connexin-43, gap-junction marker, and Nkx 2.5, transcription factor maintained during the developing step and in the adult heart, was evaluated on cardiac muscle HL-1 cells seeded on the investigated substrates to assess the capacity of the designed 3D GO–PEI hybrid material to favor the homeostasis of cardiac tissues.

## Experimental Section

### Preparation of GO and PEI Solutions

An aqueous solution of 4 g/L of graphene oxide (Graphenea, Donostia San Sebastian, Spain) was added to Ultrapure Milli-Q water (electric resistance > 18.2 MΩ/cm), from a Millipore Corp. model Direct-Q 3 system, to reach the concentration of 10 μg/mL and bath-ultrasonicated for 30 min (37 kHz, 180 W; Elmasonic P60H; Elma). The concentration of GO was checked spectrophotometrically at λ_max_ of 230 nm by using a Varian Cary 100 BIO UV–vis spectrophotometer. GO flake dimensions and ζ-potential were determined by using dynamic laser light scattering (DLS) (90Plus/BI-MAS ZetaPlus multiangle particle size analyzer, Brookhaven Instruments Corp.). For the linear polyethylenimine (PEI) solution, 8 mg of powder (average *M*_n_ 4000, PDI ≥ 1.3, MerkGaA, Darmstadt, Germany) was solubilized in Milli-Q water acidified with HCl and the solution was stirred until it was mostly clear. Sodium hydroxide was then added dropwise until pH reached ∼7.8, and the volume of the solution was adjusted with Milli-Q water to have a final concentration of 0.08 mg/mL.

### Activation of Substrates

Two different supports, i.e., 12 mm round glass coverslips and 7 × 7 mm^2^ of silicon with native oxide on its surface, were used to perform the reactions and characterizations by different techniques, respectively. All of the employed substrates were previously washed and ultrasonicated in isopropanol and acetone. Each side of the substrates was activated with a UV–ozone lamp (PSD-UV4 Novascan UV Ozone System Base model, Novascan Technologies, Boone; NC) for 30 min to increase the hydrophilicity of the surface and to improve the covalent binding of molecules.^34^ After the activation, the substrates were dipped for 1 h in a 1 M 3-(aminopropyl)triethoxysilane (APTES) (Sigma-Aldrich, ST. Louis, MO) solution in ethanol to form the silanized derivatives and then rinsed with ethanol to remove the excess of APTES.

### Preparation of GO–PEI 3D Networks

**Figure 1:**
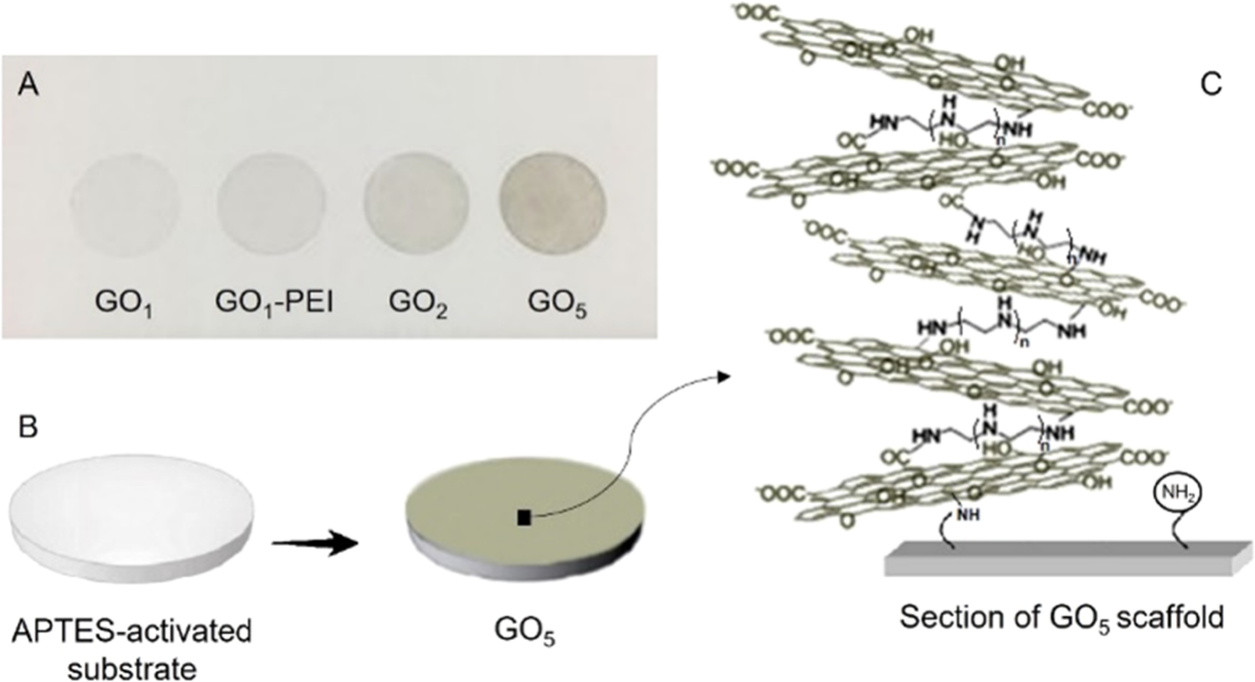
(A) Picture of the GO–PEI scaffolds obtained on an APTES-activated substrate. (B) Schematic representation of the process route with a zoom (C) on a vertical section of the GO_5_ sample surface.

The GO–PEI scaffolds were prepared by subsequently dipping the activated substrates (as above described) in aqueous solutions of GO and PEI (Figure 1). In this way, GO flakes were covalently bound to APTES amino groups, exposed on activated substrate surfaces, and PEI molecules reacted with GO through epoxy ring-opening reaction or amide formation with edge carboxylic groups of GO. The immersions of different substrates for 5 h in 10 mL of GO aqueous solution (10 μg/mL) and the dipping of the substrates in 10 mL of PEI solution (0.08 mg/mL) overnight led to the best network formation on the substrates. After each immersion, the scaffolds were rinsed with Milli-Q water to get rid of any excess of GO or PEI. Reactions were performed at room temperature. The scaffolds obtained were (i) as control, the APTES-functionalized substrate (APTES); (ii) one GO layer (GO_1_), produced by a single dipping in GO aqueous solution; (iii) one GO layer functionalized with PEI (GO_1_–PEI), manufactured by immersion of GO_1_ in PEI overnight; (iv) two GO layers (GO_2_), obtained by dipping GO_1_–PEI in GO aqueous solution; and finally (v) five GO layers (GO_5_), produced by subsequent immersion of GO_2_ in PEI, GO, PEI, GO, PEI, and GO aqueous solutions.

### SEM and AFM Characterization of GO–PEI Substrates

The morphology of GO–PEI-coated SiO_2_ substrates was investigated with a Zeiss-Gemini Leo 1530 (Zeiss, Oberkochen, Germany) field emission SEM, operating with an accelerating voltage of 5 kV, equipped with an in-lens detector. The same substrates were also characterized by AFM by using the Multimode 8 AFM microscope (Bruker, Billerica, Massachusetts) equipped with a Nanoscope V controller in an imaging mode Scan Asyst in air. Commercial silicon cantilevers RTESPA-300 (cantilever resonance frequency 300 kHz and nominal elastic constant 40 N/m) with a nominal tip radius of 8 nm were used to analyze properties such as topography across a scan size area of 10 μm × 10 μm. To measure the thickness of the networks, the incubation steps in the aqueous solutions of GO and PEI were performed by incubating only half of the surface of each substrate. Following this protocol, the surface of the substrates was half-functionalized and during the AFM analysis the area at the interface of the two parts was scanned by the probe. Moreover, the Peak Force QNM mode of AFM was used to acquire quantitative insight into the nanomechanical parameters of the GO–PEI networks, such as deformation and adhesion, as well as Young’s modulus following the Relative Method procedure given by Bruker Corporation.^35^ Toward this end, the samples were mapped using RTESPA-525 probes with a nominal spring constant of 200 N/m and a resonance frequency of 525 kHz. The deflection sensitivity of the cantilevers was calibrated against the standard Sapphire 12-M sample, while their spring constant was calculated by applying the Sader method. Highly ordered pyrolytic graphite (HOPG), with a known modulus value of 17 GPa, was used as a reference sample to calibrate the tip radius. After the calibration, the samples were scanned at a scan rate of 2 kHz, and to analyze the images, the Nanoscope Analysis 1.8 software was used.

### Raman Spectroscopy

The Raman spectra of GO–PEI-coated SiO_2_ substrates were obtained by confocal and high-performance Raman microscope (XploRA PLUS, HORIBA, Japan) with deep-cooled CCD detector technology. LabSpec (Horiba, Japan) was employed to control the Raman spectroscopic system and for the optimization and processing of the acquired data. All Raman spectroscopic measurements were performed in the range of 600–3300 cm^–1^ and with a 1800-line/mm grating. The samples were detected with a 532 nm laser, with a time of 8 s and 20 accumulations. Moderate power irradiation at the sample surface was used (∼10 mW), focusing onto the sample surface with a 50× objective to avoid laser-induced heating. For the Raman mapping of the G band intensity, an area of 100 μm^2^ was chosen by using a 100× objective and scanned with an excitation laser of 532 nm. Raman spectra reported in Figure S6 of the Supporting Information are the overlaid spectra obtained by multivariate analysis (LabSpec 6’s Multivariate Analysis module) recorded in an area of 100 μm^2^.

### Hydrophilicity

The water contact angle (CA) measurements of GO–PEI-coated glass coverslips were performed with a Krüss DSA100S instrument by depositing on GO-based films, sessile drops (2 μL) of water (5 different drops per sample). For each sample, the drop images at 1, 30, 60, and 180 s were fitted by the Ellipse (Tangent-1) method and the mean values and standard deviation were reported in Figures S7 and S8 of the Supporting Information, respectively.

### XPS Characterization

XPS analysis was employed to determine the chemical composition of the surface, thereby acquiring information on the elements as well as their chemical state, with a penetration depth of about 10 nm. XPS experiments were performed in a vacuum chamber at a base pressure of ∼10^–10^ mbar by means of a PHI-1257 (Physical Electronics, PHI) system equipped with a hemispherical analyzer and a Mg K_α_ X-ray source (*h*ν = 1253.6 eV).

### Cell Cultures

Cell cultures were set up with HL-1 Cardiac Muscle Cell Line (HL-1, SCCC065, Sigma-Aldrich, Milan, Italy). Before the establishment of cell culture, the different glass coverslips used in this study have been placed in a six multiwell coated with gelatin 0.02%/fibronectin and incubated at 37 °C in a humidified atmosphere of 5% CO_2_ in air for at least 1 h. After sucking out of the gelatin 0.02%/fibronectin, 80.000 cells/well were plated in the multiwell with Claycomb medium (51800C, Sigma-Aldrich, Milan, Italy) and placed in the incubator at 37 °C with 5% CO_2_ for 48 h.

### Study Design

All experiments were performed in triplicate with HL-1. The study design is reported as follows:

1. HL-1 cultured on a plastic bottom well, used as a negative control (CTRL);
2. HL-1 cultured on a 12 mm glass coverslip, used as a positive control (CTRL glass);
3. HL-1 cultured on a 12 mm glass coverslip treated with APTES (APTES);
4. HL-1 cultured on a 12 mm glass coverslip treated with one GO layer (GO_1_);
5. HL-1 cultured on a 12 mm glass coverslip treated with five GO layers, alternated with PEI (GO_5_);
6. HL-1 cultured on a 12 mm glass coverslip treated with one GO layer functionalized with PEI (GO_1_–PEI).

### Immunolabeling and Confocal Microscopy

The cells were fixed with 4% of paraformaldehyde in 0.1 M phosphate-buffered saline (PBS) (Lonza, Basel, Switzerland) for 45 min at room temperature, permeabilized with 0.1% of Triton X-100 for 10 min, and blocked with 5% skimmed milk in PBS for 2 h. Then, the samples were incubated with a primary mouse monoclonal antibody anti-Connexin-43 (1:200, Santa Cruz Biotechnologies, Santa Cruz, CA) and anti-Nkx 2.5 (1:200, Santa Cruz Biotechnologies) overnight at 4 °C and further incubated for 1 h at 37 °C with Alexa Fluor 568 red fluorescence conjugated (1:200, goat antimouse; Molecular Probes, Invitrogen, Milan, Italy) as a secondary antibody. Finally, all samples were incubated for 1 h at 37 °C with an Alexa Fluor 488 phalloidin green fluorescence conjugate (1:200, Life Technologies, Milan, Italy), as a marker of cytoskeleton actin and with TOPRO (1:200, Life Technologies), to stain the nuclei. The samples were monitored with a Zeiss LSM800 META (Zeiss, Jena, Germany) confocal connected to an inverted Zeiss Axiovert 200 microscope equipped with a Plan Neofluar oil-immersion objective (40x/1.3 NA). The images were collected using an argon laser beam with excitation lines at 488 nm and a helium–neon source at 543 and 633 nm. Postacquisition image analyses were carried out with a Zeiss ZEN software (ver. 2.3).^36^

### Quantitative Analysis

Five slices were evaluated from each sample to acquire data for the quantitative analysis of connexin-43 expression. Observation fields were selected according to the cell’s immunopositivity. “Multipoint” function (image processing program, National Institute of Health, Bethesda, MD) was performed for the analysis.

### Western Blot Analysis

Proteins (50 μg) from all considered conditions were treated as previously described.^37^ Membranes were incubated for 12 h at 4 °C with primary antibodies to Connexin-43 (1:500, Santa Cruz Biotechnology), to Nkx 2.5 (1:500, Santa Cruz Biotechnology), and to β-actin (1:1000, Santa Cruz Biotechnology), used as a housekeeping protocol. Then, membranes were maintained at room temperature for 30 min with a peroxidase-conjugated secondary antibody diluted 1:1000 in 1× TBS, 5% milk, and 0.05% Tween-20. To visualize protein bands, the ECL method was used; the protein level measurements were performed by means of the Bio-Rad Protein Assay (Bio-Rad Laboratories, Hercules, CA).

### Statistical Analysis

Statistical analysis was performed by GraphPad Prism 5 software and one-way analysis of variance (ANOVA) followed by post hoc Tukey’s multiple comparison tests, which were used to evaluate the statistical differences. The value of *p* < 0.05 was set as a statistically significant value.

## Results and Discussion

### Characterization of GO Aqueous Dispersion

The concentration of the GO dispersed in water was checked spectrophotometrically; the UV–vis spectra (Supporting Information, Figure S1) showed a characteristic absorption peak at 230 nm, which can be attributed to the π–π* transitions for aromatic C–C bonds and a shoulder at 290–300 nm being the fingerprint of n−π* transitions of carbonyl groups.^38^

Preliminary DLS and ζ-potential experiments evidenced that GO dispersion comprised large-sized (average diameter of 686 ± 53.7 nm) and negatively charged (−27.66 ± 1.63 mV) flakes, with a polydispersity index of 0.35 ± 0.09, indicating the modest homogeneity in size of the material (see the Supporting Information, Figure S2).

### Surface Characterization of the GO–PEI Networks

SEM and AFM analyses made it possible to study the morphology of the graphene-based scaffolds. For these measurements, because of their higher flatness, SiO_2_ substrates were used instead of glass coverslips, to be able to better resolve graphene oxide flakes in microscopy images.

**Figure 2:**
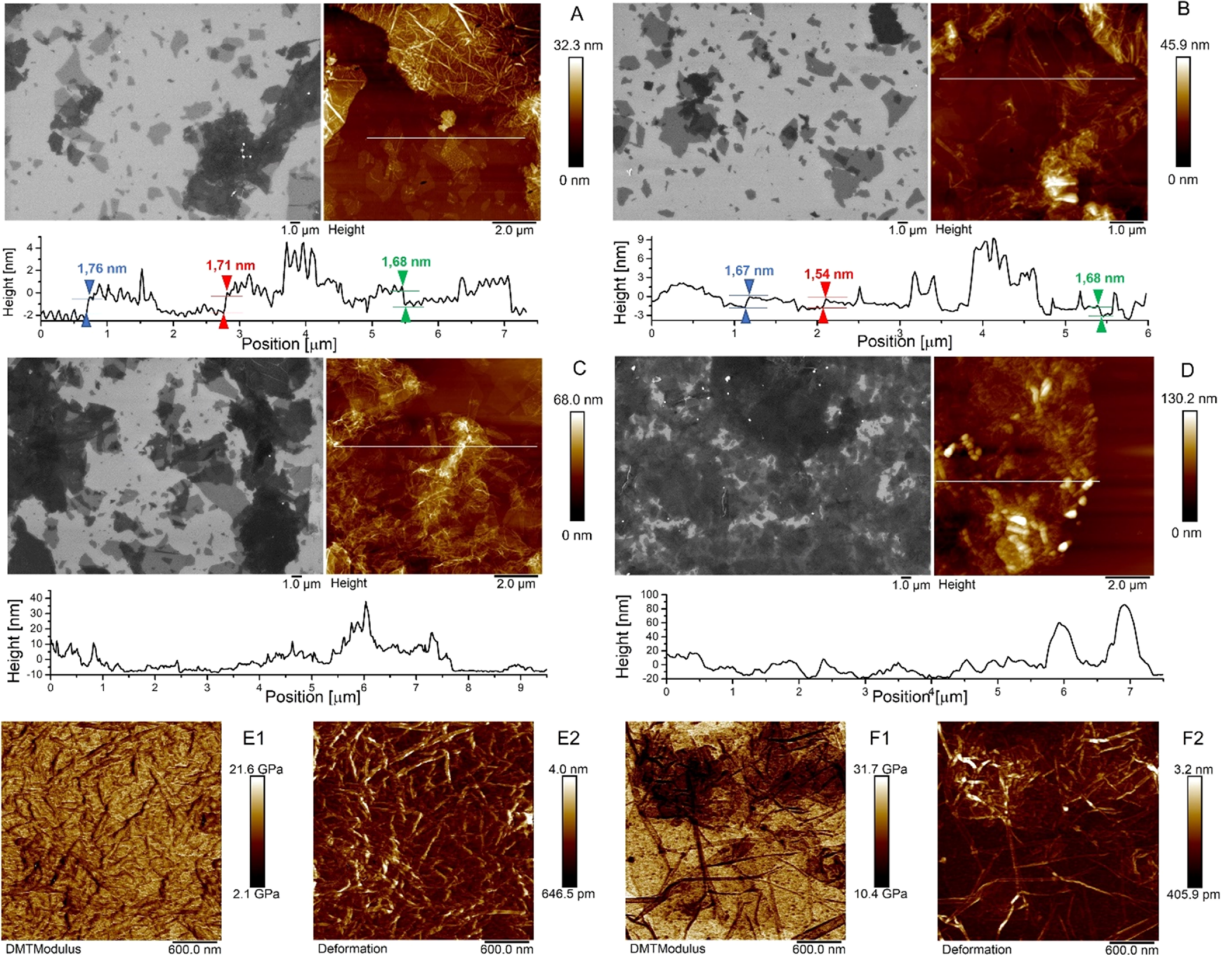
SEM micrographs and AFM images of surface topography and corresponding height cross-sectional profiles of (A) GO_1_, (B) GO_1_–PEI, (C) GO_2_, and (D) GO_5_ networks; PFQNM mode DMT modulus (E1) and deformation channels of the GO_5_ sample (E2); PFQNM mode DMT modulus (F1) and deformation channels (F2) of the GO_2_ sample.

The analyses revealed that the size of the individual sheets was not uniform, in agreement with DLS measurements. The ultrasound vibrations of the bath sonicator, during the preparation of the GO dispersion, broke the sheets into small and not homogeneous flakes. Isolated GO sheets were well visualized onto the surface of GO_1_ and GO_1_–PEI samples (Figure 2A,B), whereas upon increasing the number of incubations, isolated GO flakes could hardly be distinguished (Figure 2C,D). Indeed, GO_2_ and GO_5_ networks were characterized by large agglomerates formed by rippled GO flakes that overlap one other as connected by the PEI linkers.

In addition, the topographical AFM analysis was employed to gain insight into the three-dimensional nature of the films, thereby investigating the thickness of the different GO–PEI networks. The AFM images were analyzed by Nanoscope Analysis 1.8 Software, tracing several topographical cross sections along the GO–PEI-functionalized area and the empty substrate (see Figure 2). The height profiles of GO_1_ (Figure 2A) confirmed the presence of GO sheets with a thickness of a few nanometers in terms of vertical distance of the 3D architecture, whereas the height of each platelet amounts to ca. 1.68–1.76 nm neglecting the roughness of the flakes (see the colored arrows in Figure 2A), as reported in the literature.^39,40^ The roughness is relatively high, and this is likely due to adsorbed molecules and salts still present on the surface that could not be removed by washing as well as to overlapped GO sheets bound to the substrate through APTES spacers. On the same sample, it was also possible to notice brighter ripples with a thickness that reached values of several tens of nanometers due to the GO flakes that randomly reacted with the APTES-activated surface of the substrates and agglomerated forming thick aggregates. On the other hand, the GO_1_–PEI network (Figure 2B) displayed slightly greater thickness of the 3D architecture compared to the GO_1_ sample due to the presence of the amine PEI linkers, despite each overlapping GO sheet amounting to ca. 1.54–1.68 nm (see the colored arrows in Figure 2B). In the GO_2_ and GO_5_ samples (Figure 2C,D, respectively), the GO–PEI networks uniformly covered the surface of the substrates yielding 3D architectures characterized by a maximum thickness of 70 and 130 nm, respectively. The average thickness of the GO–PEI samples was calculated considering 80 different topographical profiles for each network. The progressive aggregation was manifested by the average thickness, calculated on 80 different points, resulting in (10.21 ± 6.13), (12.87 ± 8.50), (24.11 ± 9.58), and (46.29 ± 19.26) for GO_1_, GO_1_–PEI, GO_2_, and GO_5_, respectively. The results suggested that the thickness increased proportionally with the number of incubations.

### Determination of Young’s Modulus of the Networks

The peak force QNM mode study offered a quantitative mapping of the nanomechanical properties of the samples by quantifying indicators like the deformation and Young’s elastic modulus. It is well known that GO exhibits some unique properties that are distinctly different from pristine graphene. For example, graphene possesses Young’s modulus of ∼1.0 TPa, while the mechanical properties of GO can vary depending on the oxygen content, the thickness, and the degree of functionalization. Generally, monolayer GO has a mean Young’s elastic modulus of 250 ± 150 GPa^41^ that tends to decrease with the functionalization due to the interference of the functional moieties grafted on the *sp^3^* carbons. If one considers the GO as a rigid material, stiff cantilevers are required to apply sufficient force to indent the samples. For the measurements of the as-prepared hard materials, we used RTESPA-525 as the probe and calculated the elastic modulus by using the Derjaguin–Muller–Toropov (DMT) model and the relative method of calibration.

For the GO_5_ sample, a mean value of Young’s modulus of 12.9 ± 3.1 GPa was recorded, as shown in Figure 2E, being able to achieve a deformation of 1.99 ± 0.57 nm, consistent with the deformation obtained for the reference HOPG film (i.e., 1.69 nm). The obtained value was very low if compared to that of the GO monolayer, but it agrees with Young’s modulus obtained for multilayered GO, such as GO paper (13.6 GPa)^42^ and functionalized GO paper (16.8 GPa).^42^ Interestingly, for the sake of comparison, Young’s modulus for Nylon amounts to 1.49 GPa. Young’s modulus was found to increase with the reducing number of layers; GO_2_ was characterized by increased stiffness and decreased capacity to be deformed by the probe, showing a mean value of elastic modulus of 20.9 ± 4.4 GPa and an average deformation of 1.30 ± 0.38 nm (Figure 2F). During the analysis of the other samples, i.e., GO_1_–PEI and GO_1_ networks, it was not possible to reach the requested deformation adjusting the peak force setpoint. Figures S3 and S4 of the Supporting Information report the DMT modulus and the deformation channels for GO_1_–PEI and GO_1_ networks, respectively, obtained by increasing as much as possible the force on the samples without causing the tip wearing out or the damage of the networks. With the maximum peak force setpoint applied, we measured values of deformation of 0.42 ± 0.56 and 0.33 ± 0.39 nm for GO_1_–PEI and GO_1_ networks, respectively. The low deformation values and the related high standard deviations obtained could be a clear sign that the networks under consideration were too stiff to be indented by the tip RTESPA-525, overcoming the limit of detection of 100 GPa for this type of cantilever. However, it is important to note that as the layers decrease in the different samples, it was more difficult for the tip to indent the samples and therefore progressively less deformation was obtained. Despite the fact that data could not be properly compared with the reference, the PFQNM analysis confirmed that Young’s modulus of the networks decreased with the increase of the number of layers of GO–PEI and the degree of functionalization of GO, ranging from a nominal value of ∼250 GPa for GO monolayer to ca. 13 GPa for the scaffold with five incubations of GO (see the Supporting Information, Figures S3 and S4).

### Raman Measurements

To confirm the distribution of GO on the different functionalized substrates, Raman spectroscopy was also performed. Figure S5 of the Supporting Information reports the Raman spectrum of commercial GO, whereas Figure S6 of the Supporting Information reports the Raman spectra of the GO–PEI-coated SiO_2_ substrates. The Raman spectra confirm the presence of GO displaying D and G peaks centered at ∼1350 and ∼1600 cm^–1^, respectively, in all of the samples and, in the most homogeneously covered substrates, the presence, in the second-order region of the spectra, of the peaks at ∼2670 cm^–1^ (2D band) and 2930 cm^–1^ (combination band). Raman mapping confirms an increase of the GO flake content on increasing the number of alternative immersions in the aqueous solution of GO and PEI (see the Supporting Information, Figure S6).

### Hydrophilicity

The hydrophilicity of the surface was measured by using contact angle (CA) measurements. Figures S7 and S8 in the Supporting Information report angles measured at the drops kept at the investigated sample surface. The water contact angle at 180 s of the control glass coverslip of 70.5(±1.7)° is indicative of a glass surface, which exposes only a few hydrophilic groups on the surface. The water contact angle of 66.3(±1.5)° at the APTES surface showed an increase of hydrophilicity due to the presence of the amine groups of APTES covalently bound to the glass coverslip in perfect agreement with literature data.^43^ The GO_1_ sample presents a similar contact angle, 68.3(±2.1)°, because, despite the fact that the carboxylic acid and epoxy moieties of GO contribute to the hydrophilicity of the surface, the coverage of the surface with GO is not complete (see SEM and AFM measurements). For GO_1_–PEI, the amine and ammonium groups of PEI favor increased water affinity and therefore a further decrease of CA to 59.9(±1.2)° was monitored. For GO_2_ and overall GO_5_ samples, contact angles of 55.2(±1.7)° and 47.4(±0.8)° are displayed, respectively. These values indicate that, despite the fact that the density of hydrophilic groups on the GO surface is the same, on passing from GO_1_, GO_2_ to GO_5_, there is an increase in the coverage of the glass, as evidenced by SEM and AFM measurements. The latter values are in agreement with literature values for surfaces covered with the GO film^44^ and highlight that the presence of hydroxyl and carboxyl functional groups of GO on the basal planes contributes to the hydrophilicity and favors water filtration through the composite sample.

### XPS Analysis

**Figure 3:**
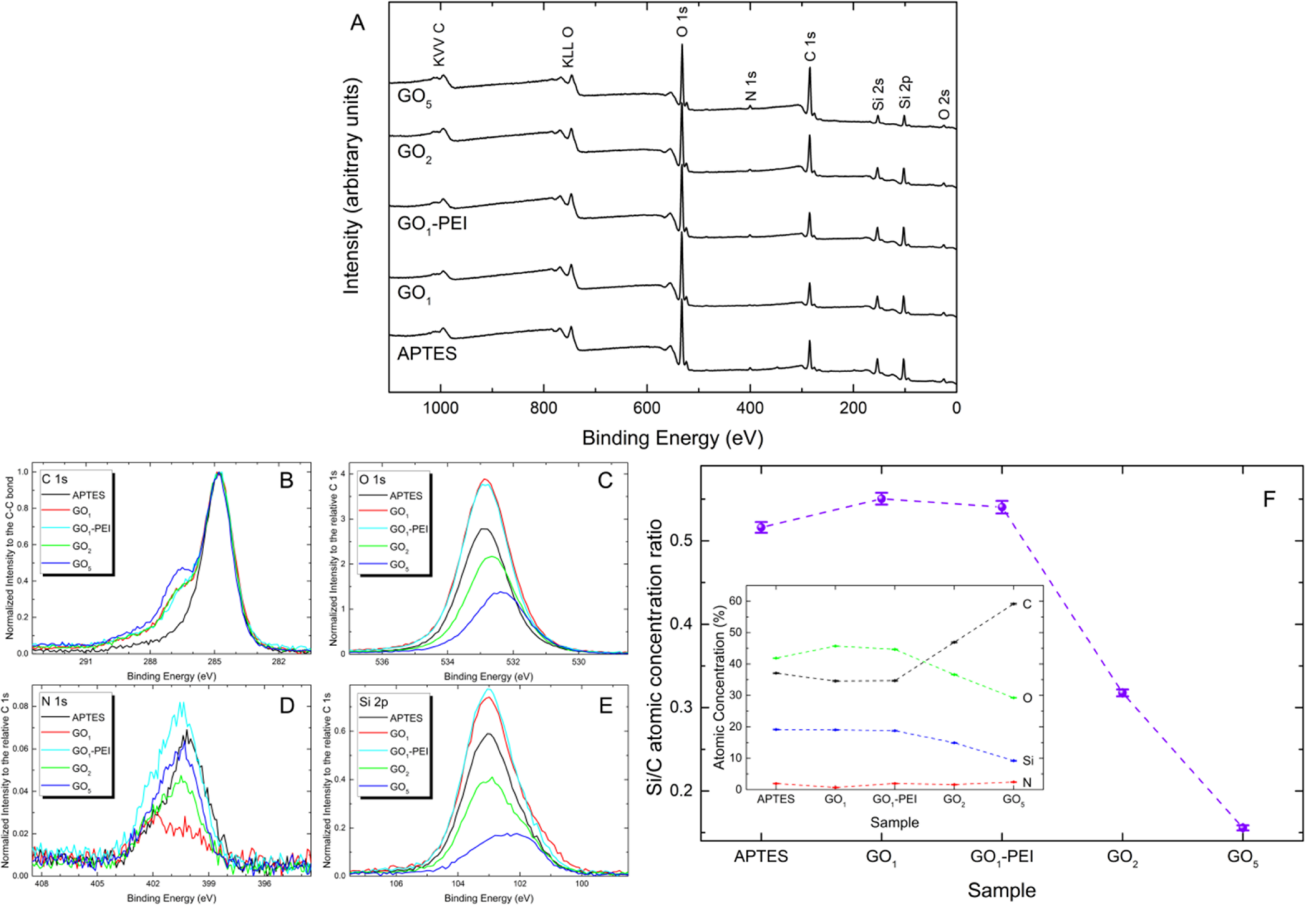
(A) XPS survey spectra of APTES, GO_1_, GO_1_–PEI, GO_2_, and GO_5_ networks. Detailed XPS scans of (B) C 1s, (C) O 1s, (D) N 1s, and (E) Si 2p of APTES, GO_1_, GO_1_–PEI, GO_2_, and GO_5_ samples. (F) Atomic concentration Si/C ratio for APTES, GO_1_, GO_1_–PEI, GO_2_, and GO_5_ samples. Inset: atomic concentrations of the same samples.

**Figure 4:**
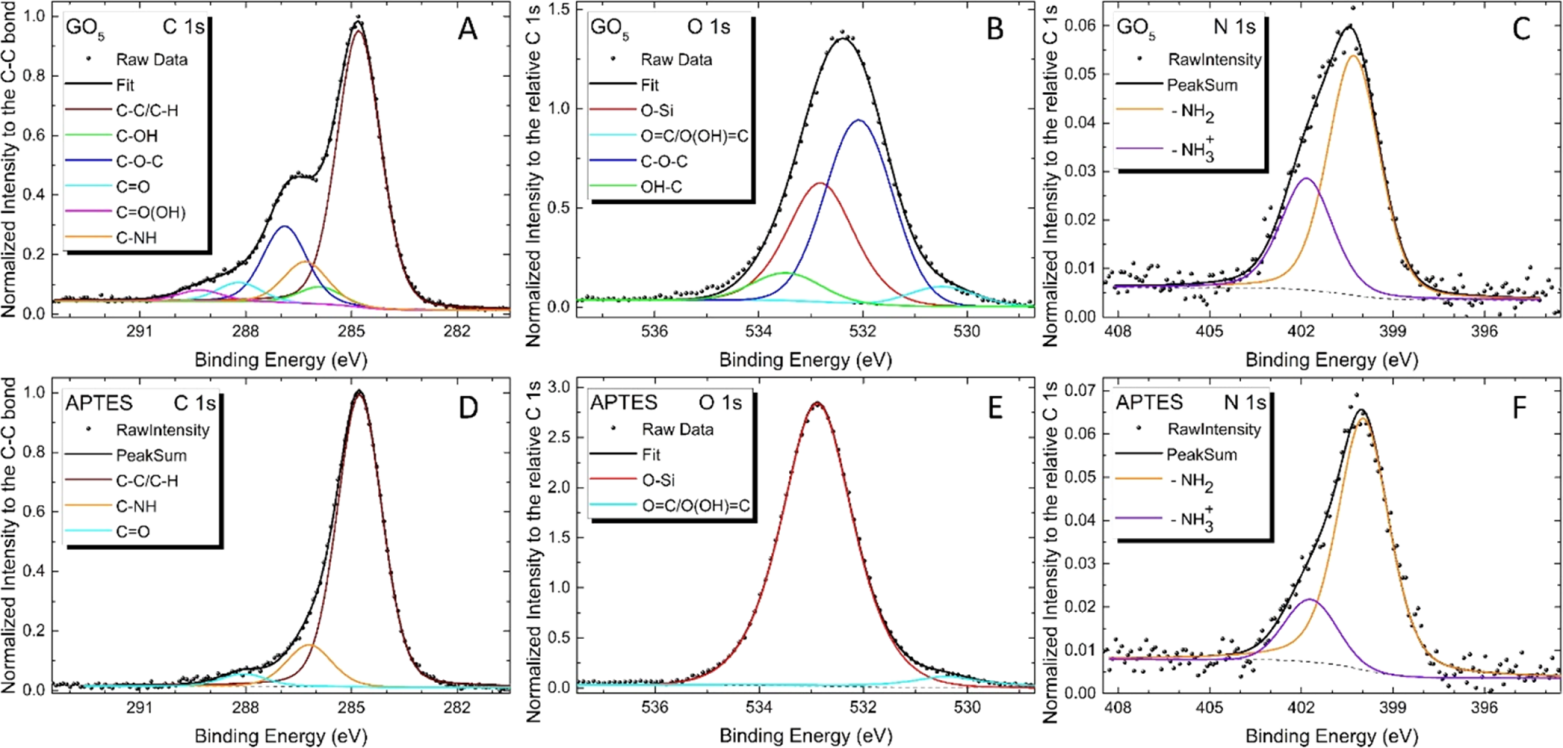
Deconvoluted XPS spectra of (A, D) C 1s, (B, E) O 1s, and (C, F) N 1s for the GO_5_ and APTES samples.

XPS analysis was surveyed in the binding energy (BE) range from 0 to 1100 eV, over 0.8 × 2.0 mm^2^ area, as shown in Figure 3A. The survey scan (Figure 3A) revealed the presence of O, C, N, and Si, all being characteristic elements present in the analyzed samples. No contamination species were observed within the sensitivity of the instrument. It is interesting to note that as a function of the increase in GO layers, starting from the APTES sample, there is a corresponding decrease in the signal of the O 1s and Si 2p peaks with an evident increase in the intensity of the C 1s peak. High-resolution core level spectra, for the identified elements, were acquired with a pass energy of 11.85 eV, corresponding to an overall experimental resolution of 0.75 eV, and the BE calibration of the spectra was referred to the C 1s peak located at BE = 284.8 eV.^45^ In Figure 4, the detailed spectra of the C 1s, O 1s, N 1s, and Si 2p peaks from the different samples are shown.

To help the reader directly visualize the comparison of the different samples, in Figure 3B, all C 1s intensities, for all of the different samples, were normalized to the C–C bond intensity, and each element (O, N, and Si shown in Figure 3C–E, respectively) detected on the sample had its own intensity normalized to the relative C 1s. Figure 3B reveals that the line shape of the C 1s peak has a shoulder at about 287 eV, indicative of the increase in the content of GO flakes due to the increase in the number of immersions in the aqueous solution of GO and PEI in sample preparation. This evidence is also confirmed by the observation of Figure 3C,E in which a decrease in the O–Si bond and a relative decrease in the intensity of the signal due to Si 2p, both caused by the effective increase in the substrate coverage, can be seen.

From the same detailed spectra, we have determined the relative concentrations of the various components. A typical expression for determining the atom fraction of elements in a sample, ***Q***_***i***_, is given by
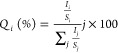
where ***I***_***i***_ and ***S***_***i***_ are the peak area and the sensitivity factor of the ***i*** element, and ***j*** is the number of different elements on the sample surface. The inset of Figure 3F reports the atomic concentrations (%) obtained from the detailed XPS spectra using the following sensitivity factors: 0.296 for C, 0.733 for O, 0.499 for N, and 0.368 for Si.

The decrease of the silicon signal, displayed in the inset of Figure 3F, on the progressive deposition of layers of GO flakes and PEI linkers from GO_1_ to GO_5_ samples, confirms the increase of the thickness of the GO–PEI scaffolds. Furthermore, the atomic concentration Si/C ratio, reported in Figure 3F, confirms an increase of the carbon signal from GO_1_ to GO_5_ in agreement with the growing number of GO layers accompanied by an increase in the coverage of the substrate under consideration.

Considering that the XPS is very useful to determine the chemical state of the identified element, all C, O, and N peaks of the APTES and GO_5_ samples have been deconvoluted by means of the Voigt multipeak and a Shirley background, keeping constant, within the experimental error, their binding energy position and full width at half-maximum.

The only variable quantity is their relative intensity. In this way, it is possible to understand which interactions are established between the GO sheets and the PEI during the different phases of the GO–PEI scaffold production process.

Figure 4A displays the spectrum C 1s that is the result of convolution of five components assigned to C atoms belonging to aromatic ring carbons (C=C/C–C, 284.8 eV), hydroxyl groups (C–OH, 285.9 eV), epoxy groups (C–O–C, 286.9 eV), carbonyl groups (C=O, 288.2 eV), and carboxyl group (C=O(OH), 289.3 eV) peaks centered, which can be ascribed to the presence of the GO flakes. In addition, a sixth component, positioned at a bond energy of 286.3 eV, is needed to involve the experimental data, and it can be attributed to the C–NH bond. Correspondingly, in Figure 4B, O 1s spectra are fitted by the sum of three components: OH–C (533.4 eV), C–O–C (532 eV), and O=C (530.4 eV), also here due to the presence of GO, and a fourth peak, at a BE of about 532.8 eV, due to the substrate. Considering the N 1s XPS spectrum (Figure 4C), we found free NH_2_ (BE of about 399.9 eV) and protonated amines **NH**_**3**_^+^ (BE of about 401.8 eV).

For the APTES sample, the fitting analysis of C 1s, O 1s, and N 1s, Figure 4D–F, respectively, highlights the chemical properties produced on the silicon oxide substrate after having functionalized it with APTES. Ultimately, from the XPS analysis, we can argue that except for the APTES-functionalized substrate, the broadening of the peak intensity at a BE of ∼ 287 eV, particularly noticeable for the GO_5_ network, is due to the characteristic functional groups of the GO and the C–NH bonds.^46^

Furthermore, the enhancement in the carbon signal from GO_1_ to GO_5_ was in agreement with the increase in the number of layers that was demonstrated also by the decrease of the ratio between Si and C (see Figure 3F). A similar conclusion could be drawn from the increase in the N 1s peak at a BE of ∼400/402 eV (Figure 4D), thus confirming an ordered assembly process and an increase in the percentage of nitrogen content, consistent with the addition of the amines to the GO with increasing the number of layers, as also revealed in Figure 3F.

Finally, the detailed XPS spectra of O 1s showed a decrease of the intensity of peaks at ∼533 eV with the increasing incubations of GO and PEI solutions (Figure 4B). This evidence could be explained by considering that the functionalization by PEI macromolecules of epoxy groups of GO led to a lower percentage of oxygen content compared to carbon and nitrogen, which are also present in the PEI molecules. Moreover, it may also confirm the effective increase in the substrate coverage following the reduction of the signal associated with the APTES-functionalized substrate. Indeed, the highest content of oxygen was recorded for the GO_1_ substrate, whose surface was characterized by uncoated areas exposing not functionalized APTES molecules, rich in oxygen atoms, and oxidized GO flakes. It is noteworthy that the atomic concentration of O in GO_5_ (see the inset of Figure 3F) is nonetheless much lower than that of the APTES-functionalized substrate, thus highlighting how the increase in the number of layers led to the formation of structures composed by sandwiched GO that because of covalent functionalization with PEI is in a more reduced (lower O/C ratio) form compared to the starting material. This evidence is in agreement with data obtained by Liu et al. demonstrating that the functionalization of GO with poly(oxylalkylene)amines of two different molecular weights ensured the partial reduction of GO.^47^

### Nkx 2.5 and Connexin-43 Expression in HL-1 Cell

**Figure 5:**
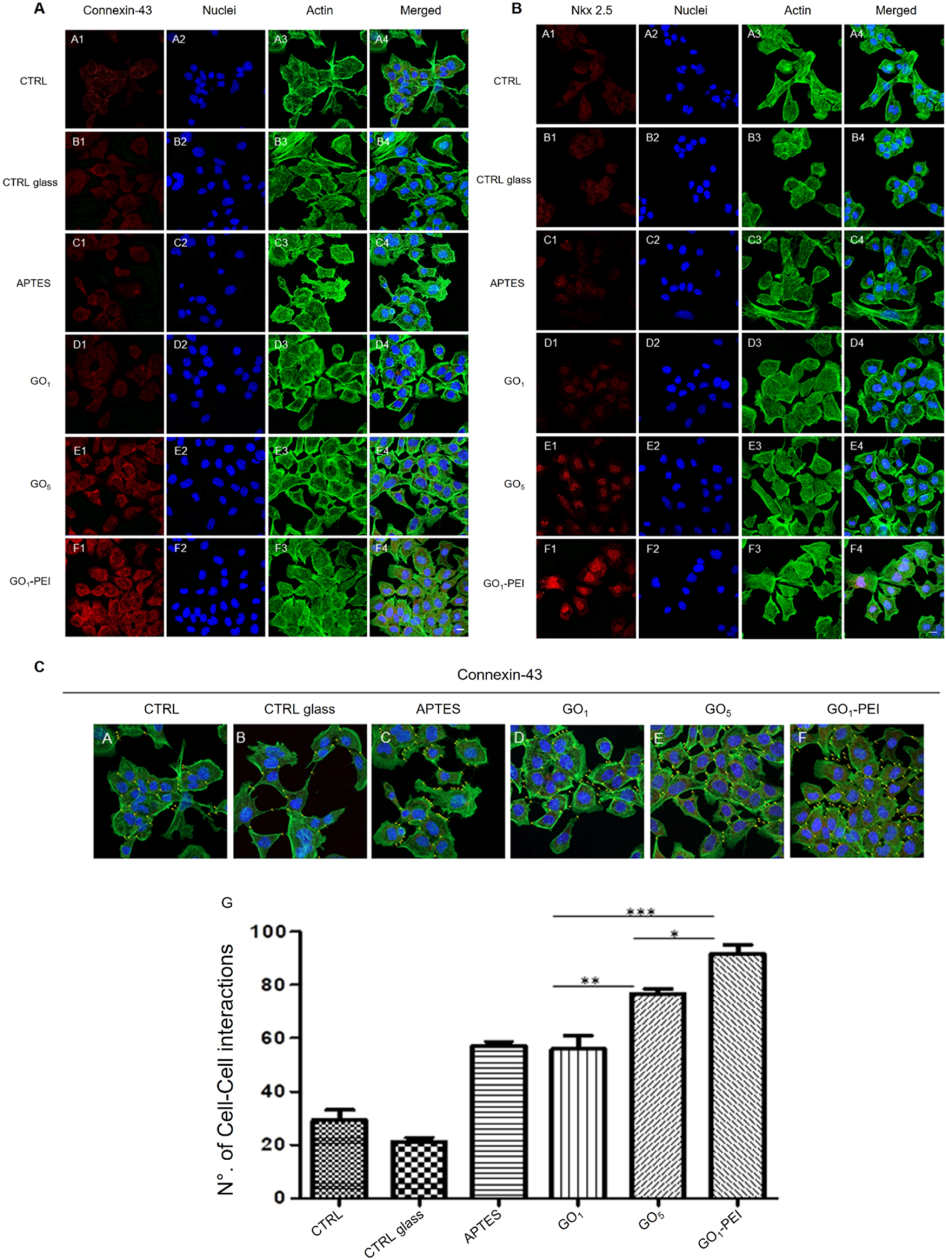
Confocal microscopy images showing the expression of Connexin-43 (panel A) and the expression of Nkx 2.5 (panel B) in HL-1 cells in all culture conditions. (Panels A and B, A1–A4) Cells cultured on a plastic substrate (CTRL). (Panels A and B, B1–B4) Cells cultured on untreated glass coverslip (CTRL glass). (Panels A and B, C1–C4) Cells cultured on APTES-treated glass coverslip (APTES). (Panels A and B, D1–D4) Cells cultured on a glass coverslip treated with one layer of GO (GO_1_). (Panels A and B, E1–E4) Cells cultured on a glass coverslip treated with five layers of GO alternated with PEI (GO_5_). (Panels A and B, F1–F4) Cells cultured on a glass coverslip treated with one sheet of GO complexed with PEI (GO_1_–PEI). (Panel C, A–F) Gap-junction distribution in HL-1 cultured cells. The expression as intracellular contact was measured (yellow dots added on images reported in panel A, A4–F4) on CTRL (A), CTRL glass (B), APTES (C), GO_1_ (D), GO_5_ (E), and GO_1_–PEI (F) confocal microscopy images. Red fluorescence: Connexin-43 or Nkx 2.5, respectively; blue fluorescence: cell nuclei, green fluorescence: cytoskeleton actin. Mag: 20×. Scale bar = 20 μm. (Panels C, histogram G) The histogram shows the means of cell-to-cell interactions for each experimental point. *p < 0.01; **p < 0.001; ***p < 0.0001.

To determine the phenotype of HL-1 and to explore the capacity of HL-1 seeded on the investigated substrates to express the cardiac markers Connexin-43 and Nkx 2.5, the immunofluorescence staining and Western blotting were performed. Cells seeded on different substrates exhibited good cell viability and the absence of morphological changes, whereas HL-1 placed on glass coverslips treated with GO_5_ and GO_1_–PEI showed a high number of focal adhesions and intercellular networks (see also the Quantitative Analysis section). The expression level of Connexin-43 and Nkx 2.5 is evident in cells plated on GO_5_ and GO_1_–PEI when compared to the other culture conditions (Figure 5, panels A and B).

**Figure 6:**
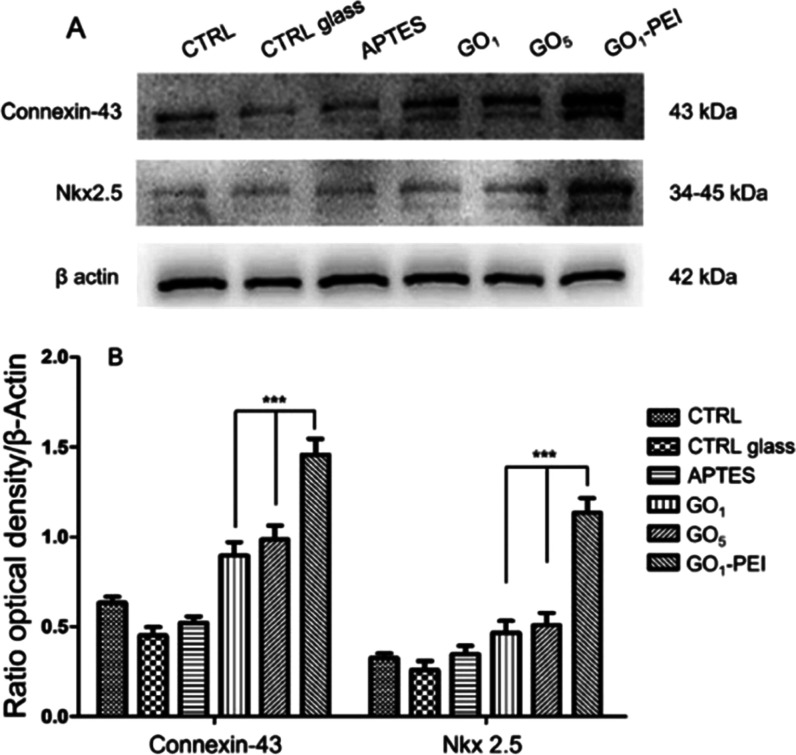
Protein expression of Connexin-43 and Nkx 2.5. (A) Protein levels of Connexin-43 and Nkx 2.5 evaluated by Western blot analysis specific bands. (B) Bar graph of band densitometric analysis normalized with β actin and housekeeping protein. Western blot is representative of three different experiments. ***p < 0.0001.

Moreover, the protein expression evaluated by means of Western blotting analysis displayed an upregulation in the expression of Connexin-43 and Nkx 2.5 in HL-1 cells cultured on GO_1_–PEI. When compared to CTRL, CTRL glass, and APTES, the Connexin-43 exhibited a greater expression level (Figure 6).

To explain the overexpression of cardiac markers of cells seeded on the GO_1_–PEI substrate, it is important to keep in mind that the initial interactions between cells and biomaterials are generally mediated by protein adsorption.^32^ The surface charge and chemical properties of the biomaterials influence surface wettability, which, in turn, govern the nature of the adsorbed protein layer. Surfaces with hydrophilic functional groups displayed higher levels of protein adsorption, which promoted cell adhesion and spreading, as demonstrated by Kumar et al.^32^ The enhanced cardiac cell organization and expression of markers involved in muscle conduction of electric signals on GO_1_–PEI could be attributed to the hydrophilic and polycationic nature of the PEI-decorated GO. Moreover, nanotopography characteristics play an important role in controlling cell behavior on biomaterial surfaces. For example, GO-coated glass was shown to provide nanoscale topographical cues for the attachment, proliferation, and differentiation of stem cells.^18^ Hence, GO–PEI scaffolds could provide the synergistic effect of GO hydrophilicity and polycationic PEI useful for cell attachment and proliferation. GO_5_ demonstrated performing better than substrates with one or two GO layers. The increased thickness of the GO–PEI scaffolds as demonstrated by AFM analyses as well as the increased percentage of reduced GO compared to the other two substrates as demonstrated by XPS analyses may favor the achievement of a 3D architecture, electrical conductivity, and the creation of a more physiologically accurate microenvironment for electroactive tissue engineering applications.^3^

### Quantitative Analysis

The most representative image of confocal acquisition of gap junction in HL-1 cells is reported in panel C of Figure 5A–F. The histogram G of panel C of Figure 5 shows the total average of the GAp43 for each experimental point. The direct cell-to-cell contacts reported in the histogram exhibit an increase in GO-coated substrates. Specifically, in cells cultured on APTES, GO_1_, GO_5_, and GO_1_–PEI, a significant increase in the number of gap junctions compared to HL-1 cultured on CTRL substrates was observed. In particular, cells seeded on GO_1_–PEI and GO_5_ demonstrated the highest increase of gap junctions, suggesting that the presence of PEI as well as the occurrence of a 3D architecture improves the formation of functional syncytia typical of cardiac organization. This organization is also favored by the association of GO with PEI.

## Conclusions

In the present study, a 3D porous scaffold composed of GO and PEI was developed for cardiac tissue engineering applications. The networks were prepared by exploiting the LbL technique with alternative incubations of APTES-activated substrate in aqueous solutions of GO and PEI, leading to the formation of scaffolds with a number of GO layers ranging from 1 to 5, separated by PEI spacers. The occurred reactions between amino groups of PEI and epoxy moieties of GO were confirmed by XPS analysis with the progressive reduction of the atomic concentration of oxygen and the increase of nitrogen content and of the C–N signal. Moreover, the increase of the carbon signal and the disappearance of the silicon signal were indicative of the increase of the layer number. The study on the morphology of the graphene-based 3D networks by AFM and SEM revealed that the functionalization of the coverslips was mostly homogeneous and that the thickness of the different samples increased proportionally with the number of incubations. Young’s modulus of the networks, measured by the PFQNM mode of AFM, decreased with the increasing number of layers of GO–PEI and the degree of functionalization of GO, in agreement with previous studies. Water contact angle measurements evidence an increase of hydrophilicity of the substrate on increasing the number of GO–PEI layers. In the biological assessment, the cardiac muscle HL-1 cells seeded on GO–PEI coverslips exhibited good cell viability, but the cells placed on GO_5_ and GO_1_–PEI substrates evidenced an upregulation in the expression of the proteins Connexin-43 and Nkx 2.5 involved in the muscle conduction of electric signals. Overall, our GO–PEI scaffolds promoted the emergence of properties required for cardiac tissue constructs such as a significant increase in the number of gap junctions compared to HL-1 cultured on CTRL substrates; hence, they hold potential for application as tissue model for drug studies or an attractive platform for cardiac tissue engineering. Further studies will be carried out to unravel the effect of the tunable pore sizes in the GO–PEI hybrid in promoting stem cell differentiation toward cardiac muscle cell lines.

## Supporting Information Available

UV–vis spectrophotometry, DLS measurements, AFM analysis, Raman spectroscopy, and contact angle measurements (PDF)

## Abbreviations

GO: graphene oxide
ECM: extracellular matrix
PEI: polyethylenimine
3D: three-dimensional
LbL: layer-by-layer
AFM: atomic force microscopy
XPS: X-ray photoelectron spectroscopy
DLS: dynamic laser light scattering
PDI: polydispersity index
CA: contact angle
CTRL: control
APTES: 3-(aminopropyl)triethoxysilane-functionalized substrate
GO_1_: one GO layer-functionalized substrate, produced by a single dipping in GO aqueous solution
GO_1_–PEI: substrate functionalized with one GO layer functionalized with PEI manufactured by immersion of GO_1_ in PEI overnight
GO_2_: substrate functionalized with two GO layers, obtained by dipping GO_1_–PEI in GO aqueous solution
GO_5_: substrate functionalized with five GO layers, produced by subsequent immersion of GO_2_ in PEI, GO, PEI, GO, PEI, and GO aqueous solutions
