# Supplementary material for: 3D Graphene Oxide-Polyethylenimine Scaffolds for Cardiac Tissue Engineering
Source: ACS Appl Mater Interfaces. 2023 Mar 7;15(11):14077–88. doi: 10.1021/acsami.3c00216 (PMC10037243; doi:10.1021/acsami.3c00216)
Supplement: Supplementary file 1 — am3c00216_si_001.pdf [file am3c00216_si_001.pdf]

SUPPORTING INFORMATION

3D Graphene Oxide-Polyethylenimine Scaffolds for Cardiac Tissue Engineering

Serena Pilato,[a] Samanta Moffa,[a] Gabriella Siani,[a] Francesca Diomede,[b] Oriana Trubiani,[b] Jacopo Pizzicannella,[c] Daniele Capista,[d] Maurizio Passacantando,[d] Paolo Samorì[e] and Antonella Fontana[*,a,f]*

[a]Dipartimento di Farmacia, Università "G. d'Annunzio" di Chieti-Pescara, Via dei Vestini, 66100, Chieti, Italy

[b]Dipartimento di Tecnologie Innovative in Medicina & Odontoiatria, Università "G. d'Annunzio" di Chieti-Pescara, Via dei Vestini, 66100, Chieti, Italy

[c]ASL02 Lanciano-Vasto-Chieti, Ospedale "Ss. Annunziata", 66100, Chieti, Italy

[d]Dipartimento di Scienze Fisiche e Chimiche, Università degli Studi dell'Aquila, Via Vetoio, 67100, Coppito, L'Aquila, Italy

[e]Université de Strasbourg, CNRS, ISIS, 8 alleé Gaspard Monge, 67000, Strasbourg, France

[f]UdA - TechLab, Research Center, Università "G. d'Annunzio" di Chieti-Pescara, Via dei Vestini, 66100, Chieti, Italy

Corresponding Author

Antonella Fontana, email: fontana@unich.it

1. UV-vis spectrophotometry page 3

Figure S1. UV-vis spectra of 10 μg/mL GO dispersion

2. DLS analysis page 4

Figure S2. DLS measurement of commercial aqueous GO dispersions. page 4

3. AFM measurements page 5

Figure S3. PFQNM mode DMT Modulus and Deformation channels of GO_1-PEI sample. page 5

Figure S4. PFQNM mode DMT Modulus and Deformation channels of GO_1 sample. page 5

4. Raman spectroscopy page 6

Figure S5. Raman spectra of commercial GO deposited onto silicon wafer. page 6

Figure S6. Cumulative Raman spectra for an area of 100 μm^2 of (A) GO_1, (B) GO_2, (C) GO_5 and (D) GO_1-PEI substrates and relevant Raman mapping of G band intensity on the same 100 μm^2 region of (E) GO_1, (F) GO_2, (G) GO_5 and (H) GO_1-PEI substrates.

page 6

5. Water contact angle measurements page 7

Figure S7. Water contact angle measurements for the investigated samples at different times. page 7

Figure S8. a) C.A. mean values for the investigated samples at different times and b) comparison of C.A. values determined at 150 s for the investigated samples. page 7

1. UV-vis spectrophotometry

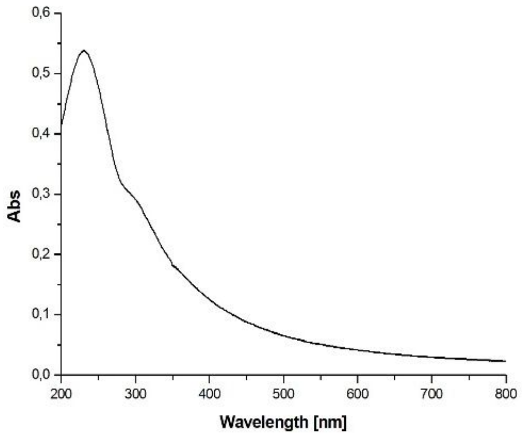

Figure S1. UV-vis spectra of 10 µg/mL GO dispersion.

2. DLS analysis

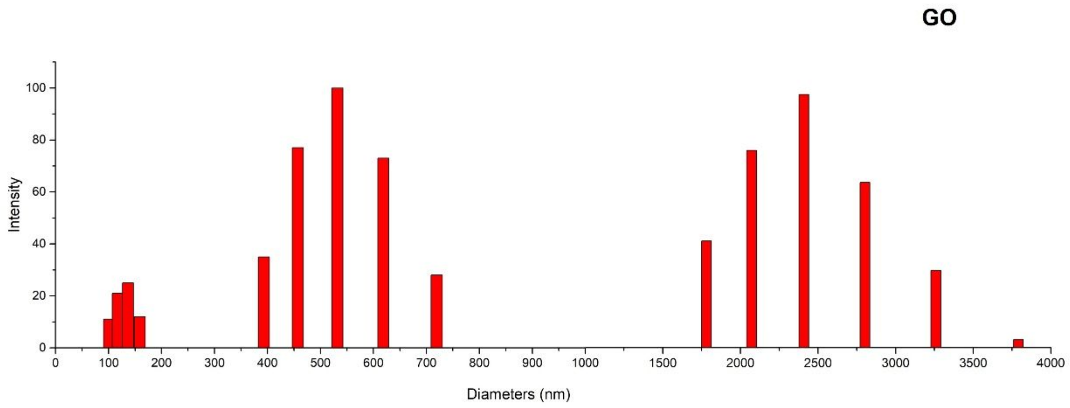

Figure S2. DLS measurement of commercial GO aqueous dispersions.

3. AFM analysis

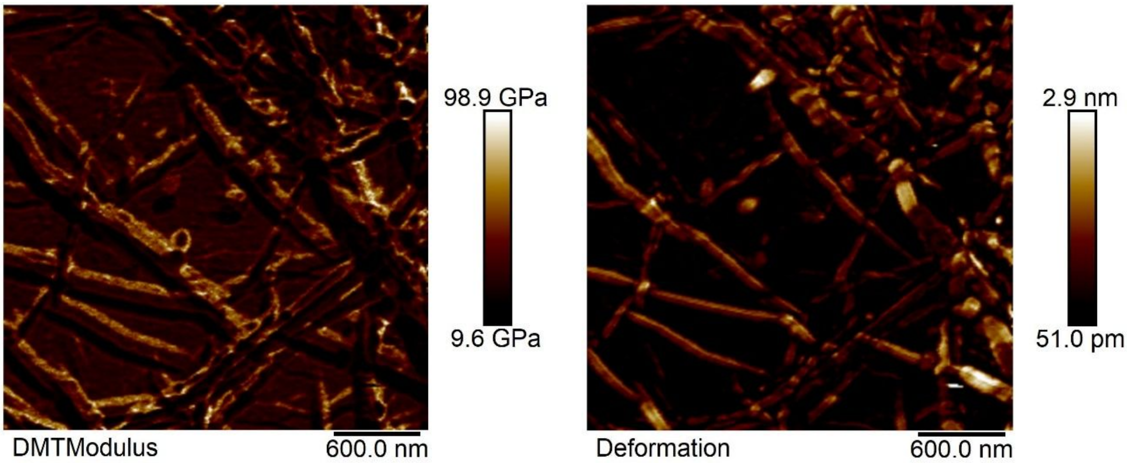

Figure S3. PFQNM mode DMT Modulus and Deformation channels of GO_1-PEI sample.

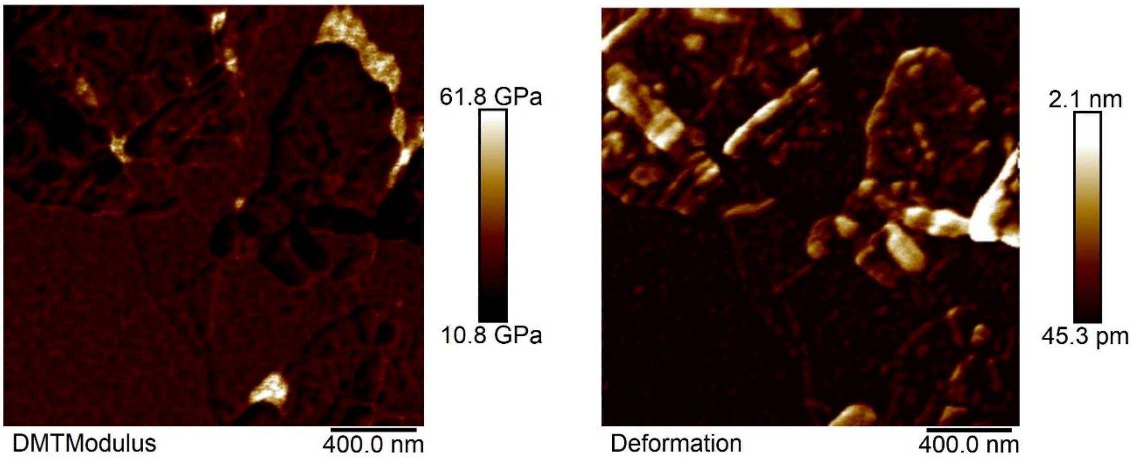

Figure S4. PFQNM mode DMT Modulus and Deformation channels of GO_1 sample.

4. Raman spectroscopy

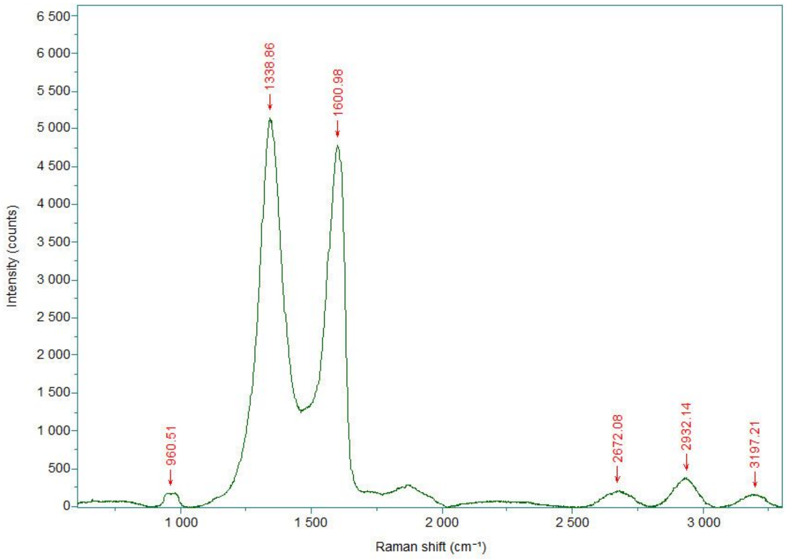

Figure S5. Raman spectra of commercial GO deposited onto silicon wafer.

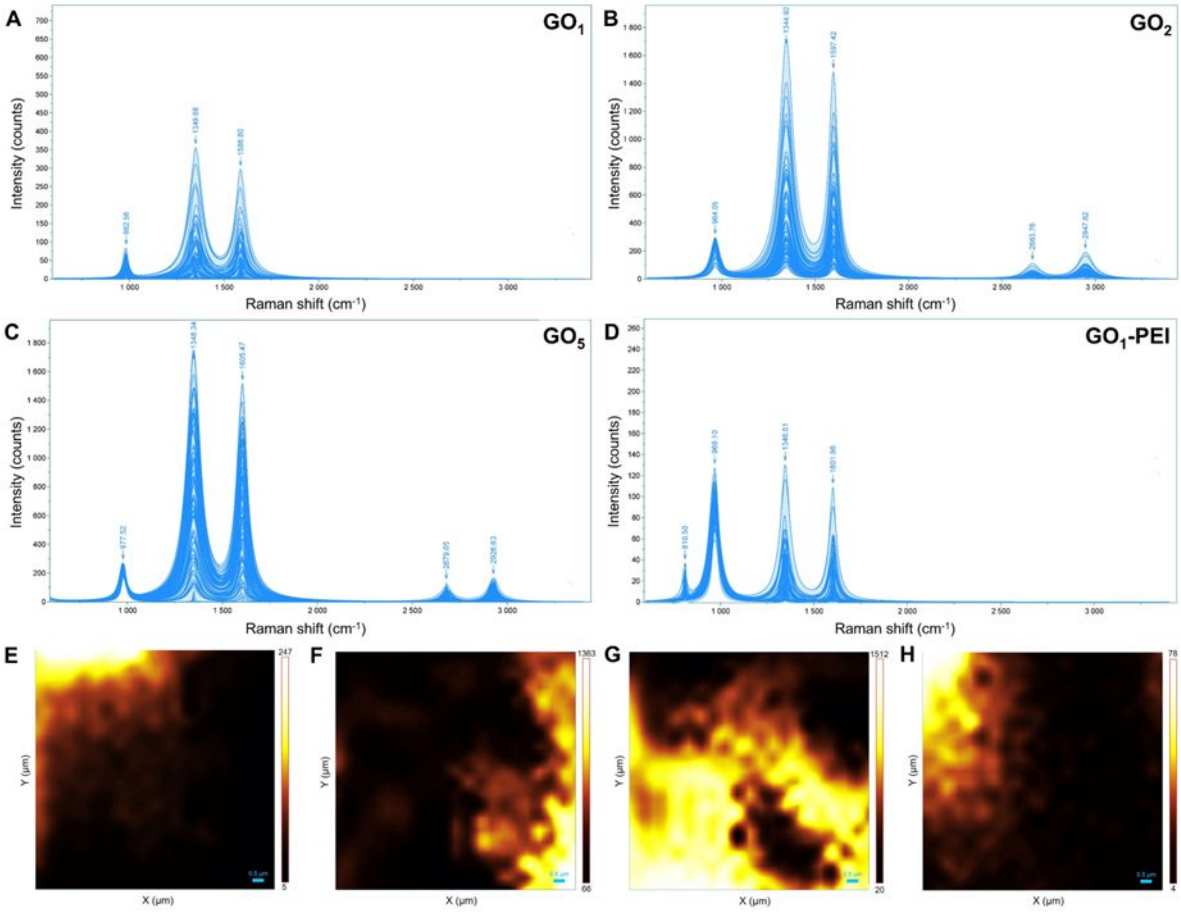

Figure S6. Cumulative Raman spectra for an area of 100 μm^2 of (A) GO_1, (B) GO_2, (C) GO_5 and (D) GO_1-PEI substrates and relevant Raman mapping of G band intensity on the same 100 μm^2 region of (E) GO_1, (F) GO_2, (G) GO_5 and (H) GO_1-PEI substrates. Scale bar 0.5 μm.

5. Water contact angle measurements

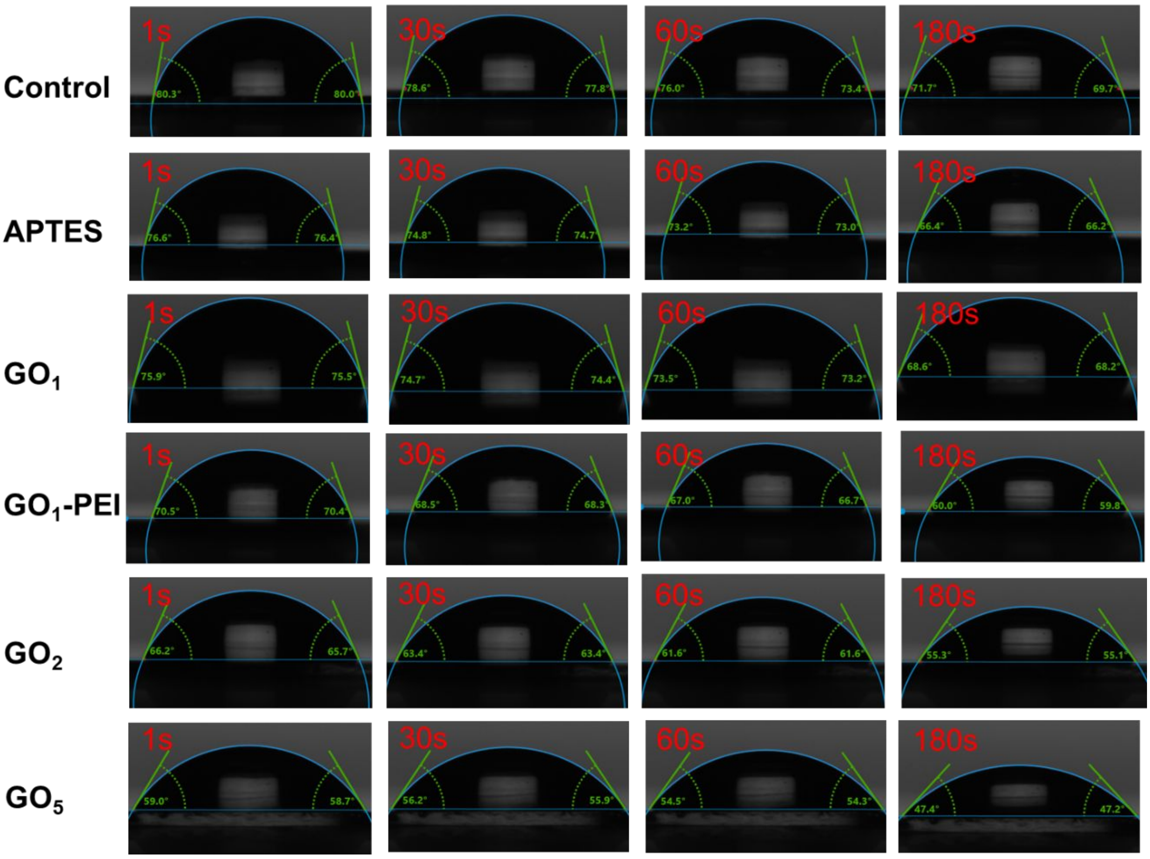

Figure S7. Water contact angle measurements for the investigated samples at different times.

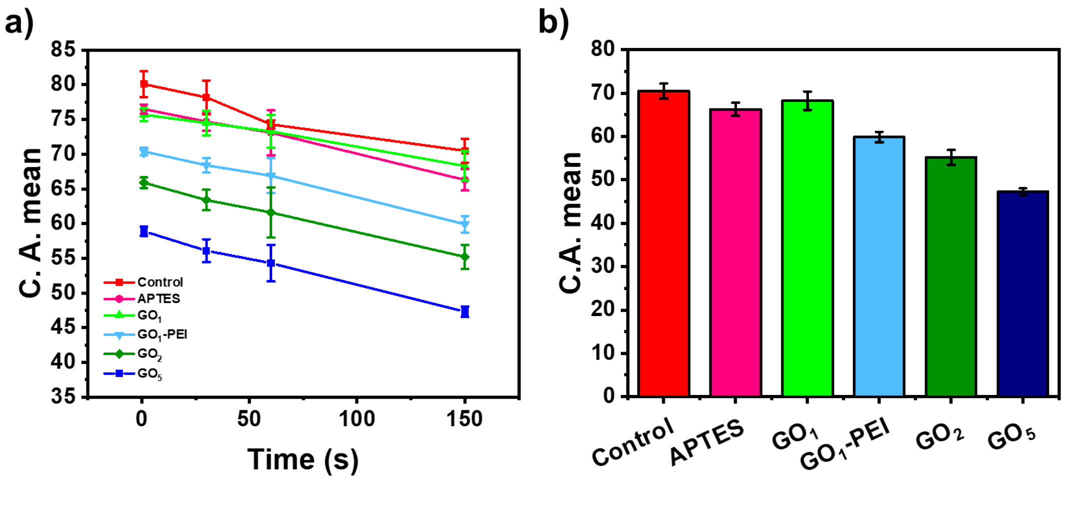

Figure S8. a) C.A. mean values for the investigated samples at different times and b) comparison of C.A. values determined at 150 s for the investigated samples.
